# Association Between Multiple Sclerosis Severity and Functional Variants in Key Antioxidant Defense and Ferroptosis-Related Genes

**DOI:** 10.3390/biology15100773

**Published:** 2026-05-12

**Authors:** Tamara Djuric, Jovana Kuveljic, Ana Djordjevic, Milan Stefanovic, Evica Dincic, Mariana Seke, Aleksandra Stankovic, Maja Zivkovic

**Affiliations:** 1 Laboratory for Radiobiology and Molecular Genetics, VINČA Institute of Nuclear Sciences—National Institute of the Republic of Serbia, University of Belgrade, 11000 Belgrade, Serbia; jovana@vin.bg.ac.rs (J.K.); ana.djordjevic@vin.bg.ac.rs (A.D.); milanst@vin.bg.ac.rs (M.S.); mariana.seke@vin.bg.ac.rs (M.S.); alexas@vin.bg.ac.rs (A.S.); majaz@vin.bg.ac.rs (M.Z.); 2 Clinic for Neurology, Military Medical Academy, 11000 Belgrade, Serbia; evica.vma@gmail.com; 3 Medical Faculty of the Military Medical Academy, University of Defense, 11000 Belgrade, Serbia

**Keywords:** multiple sclerosis, MS severity, oxidative stress, ferroptosis, gene variants, mRNA expression

## Abstract

Multiple sclerosis (MS) is a chronic, progressive, inflammatory neurodegenerative disease. Oxidative stress and ferroptosis are implicated in MS pathology, contributing to both inflammation and neurodegeneration. Potentially functional variants in genes related to antioxidant defense and ferroptosis were chosen through an extensive selection process to investigate their possible association with the progressive form of MS. The study included 845 MS patients (604 relapsing–remitting (RRMS) and 241 progressive (PMS)). The selected gene variants—*GCLC* rs572496, *GCLM* rs2273406, *GPX4* rs713041, *NQO1* rs1800566 and *CAT* rs2420388—were genotyped using TaqMan^®^ technology. mRNA expression levels of the corresponding genes in PBMCs were previously determined by targeted RNA sequencing. Circulatory molecular indicators of antioxidant defense and ferroptosis were quantified using ELISA and colorimetric enzymatic recycling assays. The findings indicate that the *GCLC* rs572496 variant was significantly associated with MS disease severity and had a significant effect on *GCLC* mRNA levels. Additionally, the *NQO1* rs1800566 variant had a significant effect on *NQO1* mRNA expression in PBMCs of MS patients overall. The results suggest further analysis of antioxidant defense and ferroptosis-related gene variants with MS severity and validation of the gained results in larger study groups.

## Simple Summary

Multiple sclerosis (MS) is a chronic condition affecting the brain and spinal cord, leading to inflammation and gradual loss of nerve function. Processes such as oxidative stress and ferroptosis (iron dependent cell death) may play an important role in how the disease develops and progresses. This study examined 845 patients with MS, including those with a relapsing–remitting form of the disease (episodes of symptoms followed by recovery) and those with a progressive form (steady worsening over time), to see whether certain gene variants are linked to more severe forms of the disease. The study included variants in the following genes: *GCLC*, *GCLM*, *GPX4*, *NQO1*, and *CAT*, and examined how these variants affect gene expression in blood cells and markers of oxidative stress and ferroptosis in the peripheral blood. The results suggest that one specific gene variant (in the *GCLC* gene) may be linked to more severe MS and changes in how the gene is expressed. Another variant (in the *NQO1* gene) was also found to influence its expression in blood cells. Overall, the findings indicate that certain gene variants may be connected to how severe MS becomes.

## 1. Introduction

Multiple sclerosis (MS) is a chronic, progressive, inflammatory neurodegenerative disease (recently reviewed in [1]). According to the WHO, MS affects more than 1.8 million individuals globally and is more frequently diagnosed in young adults and females [2]. It manifests early in life and progresses over time [3]. Due to demyelination and neurodegeneration, MS causes disability and reduces quality of life [1].

Oxidative stress is strongly implicated in MS pathology, contributing to both, inflammation and neurodegeneration [4]. It results from a disruption in the balance between reactive oxygen species (ROS) production and antioxidant defense systems, leading to cellular damage that affects both neural tissue and immune responses relevant to MS [5]. In neuroinflammatory disorders, such as MS, ROS production is excessive; thus, even the upregulation of antioxidant enzymes may be insufficient to avoid oxidative damage [5]. In addition to oxidative stress, ferroptosis, a regulated form of cell death characterized by lipid peroxidation and intracellular iron accumulation [6], has been increasingly recognized as a contributor to MS pathology [7]. It accompanies ROS buildup, leading to the deterioration of a variety of cells [8]. In MS brain tissue, macrophages and activated microglia generate significant levels of ROS, driving demyelination and promoting oligodendrocyte death, often by ferroptosis [9]. During the inflammatory phase of MS, ROS produced by macrophages and microglia cause axonal injury. The stress response activates genes that encode antioxidant and detoxifying enzymes via the Nrf2-ARE pathway [10]. The transcription factor Nrf2 maintains iron homeostasis and compensates for ROS caused damage by regulating glutathione (GSH) metabolism. Furthermore, glutathione metabolism represents a link between antioxidant defense and ferroptosis, a process involved in MS disease progression [7].

To maintain redox homeostasis and limit oxidative damage, the central nervous system (CNS) possesses intrinsic antioxidant defense mechanisms. The genes coding for the essential molecules involved in both redox balance and ferroptosis-related processes have been investigated in this study, including: (1) *GCLC* and (2) *GCLM*, subunits of glutamate cysteine ligase (GCL), an enzyme crucial and rate limiting for GSH synthesis [11]; (3) *GPX4*, an antioxidant enzyme that converts hydroperoxides from lipoproteins to the corresponding alcohols, thereby preserving intracellular redox balance and preventing the accumulation of lipid peroxides [12]. During this reduction reaction, GSH provides two hydrogen atoms and is converted into oxidized glutathione (GSSG), which can subsequently take hydrogen and be reduced back to GSH through the action of glutathione reductase (GR) in an NADPH-dependent manner [12]; (4) *NQO1*, a flavoprotein with broad antioxidant capacity that catalyzes the reduction in quinones, inhibiting their involvement in the redox cycle [13]; and (5) *CAT*, an enzyme widely expressed in CNS, primarily found in peroxisomes, where it directly eliminates ROS, since it catalyzes the decomposition of hydrogen peroxide, yielding water and molecular oxygen [10]. The strong protein–protein interactions (PPI) among these enzymes are confirmed by the String PPI enrichment value (*p* = 3.28 × 10^−12^) [14].

Common gene variants that are potential regulators of the transcription of these genes in tissues/cells of interest have been selected using publicly available eQTL databases and literature search. Considering that MS is a neurological disease with a strong inflammatory component, the focus was on variants with proposed functional effects in brain and/or blood. Selected gene variants *GCLC* rs572496 C/T and *GCLM* rs2273406 A/G may influence GSH synthesis and are proposed to exert eQTL effects in the brain and immune system, according to the GTEx database [15]. The *GPX4* rs713041 C/T variant, located in the 3′-UTR, may disrupt a region essential for the incorporation of the amino acid selenocysteine (SeCys), potentially causing premature termination of translation and the production of non-functional proteins [16]. The *NQO1* rs1800566 A/G variant results in a Pro187Ser substitution, which impairs enzyme activity ranging from reduced function to complete loss in rare allele homozygotes [13]. The *CAT* rs2420388 G/A variant has been suggested to function as an eQTL in both brain tissue and blood [15].

To date, most studies have analyzed the effect of oxidative stress on disease occurrence, but given that MS is a progressive disease, the impact of oxidative stress and related gene variants on disease progression and severity remains to be elucidated. The present study investigated potentially functional variants in antioxidant defense and ferroptosis-related genes and their possible association with a progressive form of MS.

Therefore, the aims of this study were to investigate the association of selected eQTL gene variants in antioxidant defense and ferroptosis-related genes with MS disease severity, comparing relapsing–remitting (RRMS) and progressive (PMS) MS patients; to examine if these gene variants affect target genes’ mRNA expression in PBMCs of MS patients; and to analyze their relation with circulating molecular indicators of antioxidant defense and ferroptosis.

## 2. Materials and Methods

### 2.1. Subjects

The genetic association analysis included 845 unrelated Serbian patients (604 with RRMS and 241 with PMS). Of the PMS patients, 32 had primary progressive (PP) and 209 had secondary progressive (SP) MS. A total of 222 patients (153 with RRMS, 54 with SPMS, and 15 with PPMS) made the subgroup in which potential associations of selected gene variants were analyzed with circulating biomarkers of antioxidant defense and ferroptosis. Targeted RNAseq was used to evaluate the mRNA levels in a subgroup of 48 patients (24 RRMS and 24 SPMS patients) [17].

Six hundred and twenty-three patients were recruited between 2015 and 2018 from the Clinic for Neurology of the Military Medical Academy (MMA), Belgrade, Serbia. Additionally, 222 patients who had circulatory molecular indicators of antioxidant defense and ferroptosis measured were recruited from the Clinic for Neurology, MMA and the Clinic of neurology, University Clinical Center Nis, Nis, Serbia, between 2022 and 2023. Before being included in the study, each subject provided written informed consent. The MMA Ethics Committee approved the study (Decision of 25 February 2010 and Decision No. 6/2020, approved on 4 August 2020). An experienced neurologist diagnosed each patient included in the study, in accordance with the Revised McDonald criteria [18,19,20], and a clinical approach was used to describe the course of the disease (RRMS, SPMS, and PPMS) [21,22]. A detailed questionnaire was completed for each patient using data obtained from clinical records and a neurologist-led interview, ensuring accurate collection of anthropometric and clinical data during peripheral blood sampling. Only patients with a confirmed diagnosis were included in the study. Age, sex, BMI and smoking status were among the anthropometric parameters; age at disease onset, disease duration, expanded disability status scale (EDSS), multiple sclerosis severity score (MSSS), and age-related global multiple sclerosis severity (gARMSS) score were among the clinical parameters. The EDSS score served as an indicator of neurological disability to indicate the clinical severity of the disease [23]. Disability progression was evaluated using the MSSS, which adjusts the EDSS score for disease duration [24]. For each participant, gARMSS score was calculated based on age at assessment and EDSS score. For comparing MS severity among groups based on EDSS, gARMSS offers a more versatile approach. In patients between the ages of 18 and 75, gARMSS displays the ARMSS scores derived from the EDSS [25]. An online tool was used to determine the ARMSS score [26]. Our previous papers provided a detailed description of the patients whose target gene expression was examined [17] and of the subgroup involved in circulatory biochemical analysis [27].

The main inclusion criteria were having a confirmed MS diagnosis, a minimum of one year of disease duration, and an age range of 18 to 65. Exclusion criteria included relapse within 30 days of study enrolment and, thus, recent corticosteroid treatment; clinically or radiologically isolated syndrome; comorbidities with neurological diseases other than MS and other autoimmune diseases; diagnosed cancer; pregnancy; acute infections and inability to provide informed consent. All participants reported comparable dietary habits, including regular intake of meat, vegetables, and fruit. With the exception of vitamin D, no continuous supplementation that could influence the measured parameters was reported for at least three months prior to blood collection.

### 2.2. Selection of Genes and Gene Variants

According to our previously published results [17], which applied a targeted mRNA sequencing panel comprising 138 ferroptosis-related genes, two variants were selected from two of the top differentially expressed genes (DEGs) between RRMS and SPMS patients, which were also implicated in oxidative stress pathways: *CAT* and *GCLC*. Based on a literature review and the following selection criteria, three variants were selected in the *GCLM*, *GPX4* and *NQO1* genes. The selected variants were primarily chosen for their potential functional effect on the target gene’s expression, and/or high-quality published results demonstrating their association with neurological diseases or chronic inflammatory diseases [12,28,29,30,31,32,33,34,35].

Gene variants were selected according to several criteria: (1) minor allele frequency (MAF) exceeding 0.1 in the non-Finnish European population [36]; (2) classification as an eQTL for the target gene in relevant tissues (blood, PBMCs, lymphocytes, brain) based on public databases FIVEx: eQTL [37] and/or GTEx [38]; (3) high probability of being functionally important in RegulomeDB with score at least 1f [39]; (4) GWAS gene variant–trait associations with MS identified from the GWAS Catalogue [40]; (5) information on LD with nearby variants, localization within enhancers, and the effect of variants on regulatory motifs and expression from eQTL studies, using HaploReg v4.2 [41]. The selected variants were: *GCLC* rs572496, *GCLM* rs2273406, *GPX4* rs713041, *NQO1* rs1800566 and *CAT* rs2420388 (Table 1).

### 2.3. Genetic Analysis

Genomic DNA was extracted from whole peripheral blood samples that were collected in EDTA tubes using the phenol/chloroform method. The spectrophotometric method was used to determine the quantity and quality of DNA (NanoDrop ND-1000, Thermo Fisher Scientific, Waltham, MA, USA). Samples were stored at −20 °C until genotyping. Gene variants *GCLC* rs572496, *GCLM* rs2273406, *GPX4* rs713041, *NQO1* rs1800566 and *CAT* rs2420388 were detected using the TaqMan^®^ assays for allele discrimination: C___2959429_1, C__16178733_10, C___2561693_20, C___2091255_30 and C___3102919_10, respectively, Applied Biosystems 7500 real-time PCR system and SDS software v1.4.0 (Applied Biosystems, Foster City, CA, USA). A total of 12.5 µL of TaqMan^®^ Universal PCR Master Mix, 0.625 µL of 40× TaqMan^®^ Genotyping Assay (with probes and flanking primers labeled with FAM and VIC), and 120 ng of genomic DNA were all included in the final amount of 25 µL used for PCR reactions. The probes were labeled with a reporter dye at the 5′ end and a quencher at the 3′ end, allowing allele-specific detection. Quality control measures included exclusion of samples with allele-calling rates below 95%. PCR amplification was performed under standard cycling conditions: 10 min of initial denaturation at 95 °C, 40 cycles of denaturation at 95 °C for 15 s and annealing/extension at 60 °C for 60 s. A different investigator randomly selected and genotyped approximately 10% of the samples. The results of the repeated genotyping matched those of the initial genotyping in 100% of cases. All study participants had their HLA-DRB1*15:01 status determined using the TaqMan^®^ assay C__27464665_30.

### 2.4. PBMC Isolation, Total RNA Extraction, and Targeted RNA Sequencing

PBMCs were extracted from peripheral blood using the density gradient solution Lymphocyte Separation Medium (PAA, GE Healthcare, Chicago, IL, USA). Total RNA extraction was performed using TRI Reagent^TM^ Solution (Invitrogen^TM^, Thermo Fisher Scientific, Waltham, MA, USA) within 30 min of sample collection in accordance with the manufacturer’s instructions. RNA integrity was maintained using RiboLock RNase Inhibitor (Thermo Fisher Scientific, Waltham, MA, USA) and samples were kept at −80 °C in nuclease-free water prior to use. A NanoDrop ND-1000 spectrophotometer (Thermo Scientific, Waltham, MA, USA) was used to determine the concentration and purity of RNA. Targeted RNA-seq library preparation was performed using the AmpliSeq™ Library PLUS (Illumina, Inc., San Diego, CA, USA) for Illumina^®^ protocol on PBMC RNA samples from 48 MS patients (24 RRMS and 24 SPMS), as previously described [17]. Briefly, 50 ng of total RNA was reverse-transcribed and subjected to targeted amplification using a custom-designed RNA panel of ferroptosis-related genes. Following partial digestion, adapter ligation, and purification, libraries were quantified using a Qubit 3.0 Fluorometer (Thermo Fisher Scientific, Waltham, MA, USA), normalized, diluted and pooled to a final loading concentration of 50 pM.

Targeted RNA sequencing was performed on pooled libraries using the iSeq™ 100 System with a 2 × 151 bp run configuration. Quality control included monitoring of sequencing metrics and on-target read distribution. Following demultiplexing and alignment to target regions, raw read counts were generated. Differential expression analysis and normalization were performed using the DeSeq2 workflow, as described in our previous study [17]. Normalized read counts for selected genes (*GCLC*, *GCLM*, *GPX4*, *NQO1*, and *CAT*) were used for downstream analyses in the present study.

### 2.5. Quantification of GPX4, 4-HNE, GSH, GSSG and Total Glutathione in Plasma

Peripheral blood samples taken in EDTA tubes were centrifuged at 1000× *g* for 15 min at 4 °C within 30 min after collection. Plasma was collected and kept at −20 °C prior to use. Human GPX4 (Phospholipid hydroperoxide glutathione peroxidase 4) and 4-HNE (4-Hydroxynonenal) were quantified in the plasma of 222 patients (153 with RRMS, 54 with SPMS and 15 with PPMS) using the Fine Test^®^ ELISA kit (Wuhan Fine Biotech Co., Ltd., Wuhan, China), according to the manufacturer’s instructions, as previously described [21]. Optical density (OD) was measured using the PKL PPC 142 ELISA reader (PKL^®^ Pokler Italia, Paramedical srl, Salerno, Italy) at 450 nm. The mean of duplicate OD readings for each sample was used to determine GPX4 (pg/mL) via a Four Parameter Logistic (4PL) curve [42].

Levels of oxidized (GSSG), reduced (GSH), and total (GSSG + GSH) glutathione were measured in plasma samples from 100 MS patients, 63 of whom had RR and 37 of whom had PMS, using a quantification kit for oxidized and reduced glutathione (Sigma-Aldrich^®^, Merck KGaA, Darmstadt, Germany). GSSG and total glutathione were quantified using a colorimetric enzymatic recycling assay based on the reaction with 5,5′-dithiobis-(2-nitrobenzoic acid) (DTNB). Free GSH in the sample was masked by the addition of a masking reagent provided in the kit. Subsequently, GSSG was reduced to GSH by glutathione reductase, and the resulting GSH reacted with DTNB to form a yellow-colored product (TNB), which was measured spectrophotometrically at 412 nm using a PKL PPC 142 ELISA reader (PKL^®^ Pokler Italia, Paramedical srl, Salerno, Italy). The concentration of glutathione (μmol/L) is directly correlated with the rate of TNB formation. GSH concentration (μmol/L) was calculated by subtracting GSSG from total glutathione, in accordance with the manufacturer’s instructions.

### 2.6. Statistical Analysis

Genotype and allele frequency distributions, categorical variables, and deviation from Hardy–Weinberg equilibrium were all compared using the chi-square (χ^2^) test. Logistic linear regression was performed to estimate the strength of association between genotypes and the phenotype of interest, with results presented as odds ratios (ORs) with corresponding 95% confidence intervals (CIs). The PS-Power and Sample Size calculator was used to estimate the statistical power of the study for the observed associations between gene variants and the phenotype [43]. The normality of continuous variables was assessed using the Kolmogorov–Smirnov test with Lilliefors correction and the Shapiro–Wilk test. Continuous variables were compared between the two groups using either Student’s *t* test or the Mann–Whitney U test, according to their distribution (normal or skewed, respectively). To make the data more meaningful for the readership, it was presented as mean ± standard deviation (SD). All statistical analyses were conducted using Statistica 8.0 (StatSoft, Inc., Tulsa, OK, USA). *GCLC*, *GCLM*, *GPX4*, *NQO1* and *CAT* mRNA expression levels were presented as log_2_-transformed read counts. Graphical data presentation was performed using GraphPad Prism v6.01 (GraphPad Software, Inc., Boston, MA, USA). The correlations of GPX4 with circulatory molecular indicators of glutathione-related antioxidant defense (GSH, GSSG, GSH/GSSG ratio) were estimated with Spearman’s rank-order correlation test. In all analyses, *p* values below 0.05 were considered statistically significant. Multiple linear regression analysis was performed to evaluate whether the *GCLC* rs572496 and *NQO1* rs1800566 were associated with target gene mRNA expression independently of confounding factors (age, sex, disease course, disease duration, EDSS, MSSS and gARMSS), and presented as β ± SE coefficient with *p* value. The Post-hoc Power Calculator was used to assess the statistical power of the gene expression analyses [44]. Partial Spearman correlation analysis between GPX4 and circulating molecular indicators of glutathione-related antioxidant defense was performed using IBM SPSS Statistics 20.0 (IBM Corp., Armonk, NY, USA, 2011).

## 3. Results

### 3.1. Genetic Association Analysis of Selected Gene Variants with MS Disease Severity

The basic characteristics of the study groups included in the genetic association analysis with MS severity are presented in Table 2. This analysis of the study groups was previously published in Djuric et al., 2025 [45]. Patients with PMS were older than RRMS patients, and had higher EDSS, MSSS and gARMSS scores, as well as longer disease duration. Smoking status was similarly distributed between RRMS and PMS patients (Table 2).

These differences reflect the well-established natural course of multiple sclerosis, in which RRMS patients are typically younger, while the progressive form occurs later. As expected, due to disease progression, PMS patients had a longer disease duration and higher EDSS score, MSSS and gARMSS [3].

The frequency distribution of *GCLC* rs572496 C/T genotypes differed significantly between RRMS and PMS (χ^2^ test, *p* = 0.02). The frequency of the rare allele homozygote TT was significantly lower in PMS patients compared to RRMS (χ^2^ test, *p* = 0.007) (Table 3). This result shows that TT genotype has a protective effect against the severe form of MS with an adjusted OR = 0.52, 95% CI = 0.32–0.84, *p* = 0.007. The OR was adjusted for the HLA-DRB1*15:01 rs3135388 variant because it represents one of the most important sources of genetic confounding in MS studies [46]. The study power of 84% supports the validity of this association. When the *GPX4* rs713041 variant was analyzed between RRMS and PMS patients, it was found that rare T allele had a protective effect against the progressive MS, but only in male MS patients (OR = 0.64, 95% CI = 0.45–0.92, *p* = 0.02) (Table 3). The sex-specific associations are not novel [47], but since the power of the study was 64%, this result must be validated in larger groups. The other three investigated gene variants, *GCLM* rs2273406, *NQO1* rs1800566 and *CAT* rs2420388, did not show a significant association with MS severity (Table 3). GCLM has the lowest MAF, and this may be because we did not have enough study power on this sample size to reach the statistical significance.

Analysis of the possible associations between the investigated variants and MS neurological deficit and disease severity parameters (EDSS, MSSS, and gARMSS) did not show any significant associations. Although these variants are involved in MS pathology, they are not independently related to clinical parameters of MS progression. Having in mind the polygenic nature of MS severity and the fact that it is still not well investigated, the results suggest further investigation of other gene variants that could bear predictive risk for this phenotype.

### 3.2. Effects of GCLC rs572496, NQO1 rs1800566, GCLM rs2273406, GPX4 rs713041 and CAT rs2420388 on Their Relative mRNA Expression in PBMCs of MS Patients

**Figure 1:**
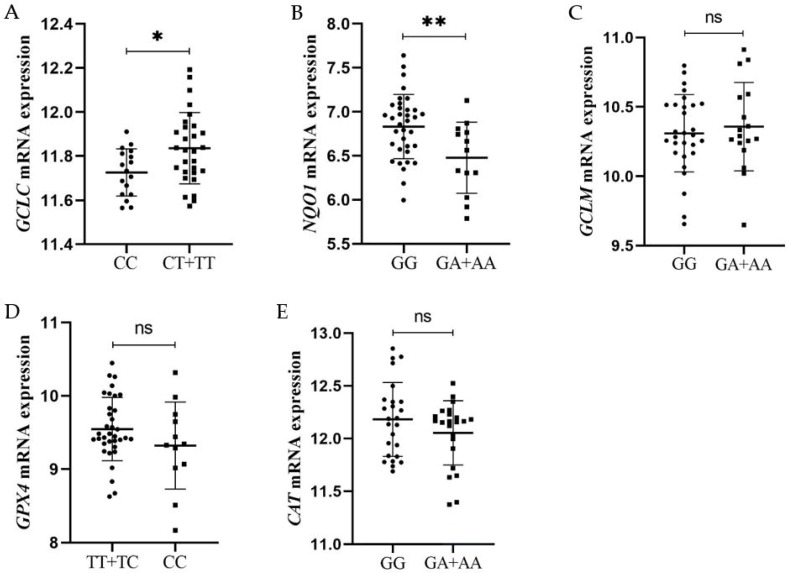
Association of investigated gene variants with relative mRNA expression in PBMCs from MS patients. Relative mRNA expression was measured by targeted RNAseq and is presented as read counts for each sample (n = 48). Data are presented as log_2_ transformed read counts with mean for both groups ± SD. Differences in relative mRNA expression between groups were assessed using either Student’s *t* test or the Mann–Whitney U test. (**A**) Relative expression of *GCLC* mRNA according to the rs572496 dominant model (CC, circles vs. CT + TT, squares). A significant upregulation of *GCLC* mRNA was detected in PBMCs from carriers of the T allele (n = 29) compared to carriers of the CC genotype (n = 17) (11.835 ± 0.161 vs. 11.726 ± 0.106, *p* = 0.016, Student’s *t* test). (**B**) Relative expression of *NQO1* mRNA according to the rs1800566 dominant model (GG, circles vs. GA + AA, squares). Significant downregulation of *NQO1* mRNA was detected in PBMCs from carriers of the G allele (n = 13) compared to carriers of the GG genotype (n = 35) (6.479 ± 0.404 vs. 6.832 ± 0.365, *p* = 0.006, Student’s *t* test). (**C**) Relative expression of *GCLM* mRNA according to the rs2273406 dominant model (GG, circles vs. GA + AA, squares). *GCLM* mRNA expression was not significantly different between carriers of GG genotype (n = 29) and carriers of the A allele (n = 17) (10.310 ± 0.278 vs. 10.358 ± 0.319, *p* = 0.59, Student’s *t* test). (**D**) Relative expression of *GPX4* mRNA according to the rs713041 recessive model (TT, circles + TC vs. CC, squares). *GPX4* mRNA expression was not significantly different between carriers of the T allele (n = 35) compared to carriers of CC genotype (n = 12) (9.547 ± 0.430 vs. 9.322 ± 0.594, *p* = 0.16, Student’s *t* test). (**E**) Relative expression of *CAT* mRNA according to the rs2420388 dominant model (GG, circles vs. GA + AA, squares). *CAT* mRNA expression was not significantly different between carriers of GG genotype (n = 25) and carriers of the A allele (n = 23) (12.182 ± 0.351 vs. 12.055 ± 0.305, *p* = 0.32, Mann–Whitney U test). * *p* ≤ 0.05; ** *p* ≤ 0.01. ns—non significant.

All of the selected variants are proposed as eQTLs that could affect target genes’ mRNA expression. The results show an association of *GCLC* rs572496 with relative *GCLC* mRNA levels in PBMCs from MS patients according to the dominant model (CC vs. CT + TT) (Figure 1A). Genotypes containing the rare T allele had significantly higher *GCLC* mRNA levels in MS patients compared with those carrying the wild-type homozygote CC (11.835 ± 0.161 vs. 11.726 ± 0.106, *p* = 0.016, Student’s *t* test). Even the number of patients in the expression analysis was n = 48; the study power for this association was 78.9%, which validates the result.

The other investigated variant that confirmed its effect on target gene expression was *NQO1* rs1800566. Genotypes containing the rare A allele (GA + AA) had significantly lower *NQO1* mRNA levels in PBMCs of MS patients compared with patients carrying the wild-type homozygote GG (6.479 ± 0.404 vs. 6.832 ± 0.365, *p* = 0.006, Student’s *t* test) (Figure 1B). The study power for this association was 78.8%, further validating this finding. *GCLM* rs2273406, *GPX4* rs713041 and *CAT* rs2420388 showed no significant association with their relative mRNA levels in PBMCs of MS patients (Figure 1C–E). As many confounding factors could influence mRNA expression, these findings were adjusted for age, sex, disease course, disease duration, EDSS, MSSS and gARMSS using multiple linear regression. The association of *GCLC* rs572496 and *NQO1* rs1800566 with target gene mRNA expression remained significant, supporting the independent effect of gene variants (β ± SE = 0.31 ± 0.14, *p* = 0.03 and β ± SE = −0.38 ± 0.13, *p* = 0.006, respectively) (Tables S1 and S2). Since there is a protective effect of T allele-containing genotypes toward progression of MS and significantly higher *GCLC* mRNA expression in those patients, the results suggest that this variant could lead to better antioxidant defense and a balanced redox state through its role in glutathione synthesis [11]. On the other hand, *NQO1* limits ROS production by preventing the formation of harmful semiquinone radicals [48] and patients with *NQO1* rs1800566 genotypes containing the rare A allele have lower mRNA expression. This result suggests that this variant is functional and could bear risk for neurodegenerative diseases, as reduced NQO1 protein levels have been associated with Alzheimer lesion formation [34].

### 3.3. Associations of Investigated Gene Variants with GSH, GSSG, GSH/GSSG Ratio and GPX4 in Peripheral Blood Plasma Samples of MS Patients

Plasma concentrations of GSH, GSSG, and GPX4 were measured and the GSH/GSSG ratio was calculated in a subgroup of MS patients, as a valid marker of redox state, in a subgroup of MS patients (n = 100), and their possible association with the investigated gene variants were analyzed (Table S3). None of the investigated gene variants have shown an association with glutathione-related antioxidant defense circulating markers, except *CAT* rs2420388. Carriers of *CAT* rs2420388 A allele-containing genotypes (GA + AA) had lower GPX4 plasma concentrations compared to carriers of the GG homozygote (2180.05 pg/mL ± 1218.09 pg/mL vs. 2736.80 pg/mL ± 1680.85 pg/mL, respectively, *p* = 0.03, Mann–Whitney U test). Both GPX4 and catalase decompose hydrogen peroxide into water and oxygen, so they play a role in antioxidant defense. This variant has previously been associated with lower plasma CAT levels in GWAS [49], so it could lead to higher oxidative stress.

### 3.4. Correlation of Circulatory Molecular Indicators of Glutathione-Related Antioxidant Defense and Lipid Peroxidation (GSH, GSSG, GPX4, 4-HNE) in Plasma Samples of MS Patients

Besides circulatory molecular indicators of glutathione-related antioxidant defense (GSH, GSSG, GPX4), a molecular indicator of lipid peroxidation and ferroptosis, 4-hydroxynonenal (4-HNE), was also measured [27]. The values of calculated GSH/GSSG ratio were divided into quartiles. Overall, the GSH/GSSG ratio was rather low in MS patients (mean 1.24, median 1.05 [0.76–1.55]), pointing to higher oxidative stress. Patients in the highest quartile of GSH/GSSG ratio (75th percentile, 1.55) had significantly lower plasma 4-HNE (*p* = 4 × 10^−5^), which indicates interplay between ferroptosis and oxidative stress in MS pathology.

A significant positive correlation between GPX4 and GSH (Spearman’s R = 0.30, *p* = 0.003) and the GSH/GSSG ratio (Spearman’s R = 0.34, *p* = 0.001), as well as a negative correlation with GSSG in plasma (Spearman’s R = −0.26, *p* = 0.009), were observed, suggesting that higher GPX4 levels are associated with a more favorable (less oxidative) glutathione redox state, characterized by higher GSH and lower GSSG levels (Table 4). Scatter plots with regression lines showing the observed correlations are presented in Figure S1. To address potential confounding factors, partial Spearman’s correlation analysis was performed, which confirmed that the observed associations remained statistically significant after adjustment for age, sex, disease course and disease duration.

## 4. Discussion

The present study investigated five potentially functional variants in key antioxidant defense and ferroptosis-related genes in association with MS severity. So far, studies investigating oxidative stress in MS reported higher levels of advanced oxidation protein products in plasma and cerebrospinal fluid in RRMS patients [50], and a nearly 1.5-fold decline in total antioxidative capacity levels (AOC) compared to controls [51]. In addition, lower AOC in cerebrospinal fluid showed an association with higher disability at time of sampling and with EDSS >3 scores in the early MS disease [52]. In this study, an association between MS severity and the *GCLC* rs572496 and *GPX4* rs713041 variants was observed. The findings also support a proposed regulatory effect of *GCLC* rs572496 and *NQO1* rs1800566 on target gene mRNA expression. The *CAT* rs2420388 variant was found to affect GPX4 plasma level in the entire MS group of patients, and patients in the quartile with a highest GSH/GSSG ratio had significantly lower 4-HNE, the molecular indicator of lipid peroxidation.

### 4.1. GCLC and GCLM

GCLC is the most catalytically active as a heterodimer with the GCLM subunit [53]. This study showed that *GCLC* rs572496 rare allele homozygotes (TT genotype) had a protective effect toward the progressive form of MS in investigated study groups and that rare T allele-containing genotypes were independently associated with significantly higher *GCLC* mRNA levels in PBMCs of MS patients compared to the CC homozygotes. The variant in the gene coding modifier subunit of GCL, *GCLM* rs2273406, did not reach significance in association with progressive form of MS. Rare GG genotype and G allele of another variant in *GCLC*, rs648595, which is in almost absolute linkage disequilibrium with *GCLC* rs572496 investigated in this study, were associated with protective effect toward psoriasis [28]. Both multiple sclerosis and psoriasis are chronic inflammatory diseases that include dysregulated immune systems, particularly involving T-cells and specific pro-inflammatory cytokines including TNF-α, IL-17, and IL-23, alongside genetic risk factors (HLA genes) and shared environmental triggers like obesity, smoking, and vitamin D deficiency, all leading to chronic inflammation, though they target different tissues [54]. Qi et al. have identified gene variants for brain traits from blood data by leveraging the strong correlation (0.7–0.78) of genetic eQTLs effects between blood and brain tissues, using large-scale datasets on the population with European descent. They found genes linked to brain-related phenotypes demonstrating blood’s power for discovery, especially for complex conditions where direct brain tissue access is limited. They found that *GCLC* rs572496 is eQTL highly correlated between independent brain and blood samples [29]. In this study, its effect on *GCLC* mRNA levels in PBMCs of MS patients has been confirmed. The observed protective effect of rare T allele-containing genotypes against progressive MS, together with higher mRNA expression in PBMCs of MS patients, may suggest better antioxidant defense in RRMS patients. Certainly, very important factors that can affect post-translational regulation of these molecules [55] should be taken into consideration.

### 4.2. GPX4

GPX4 is the only selenoprotein glutathione peroxidase that can reduce lipid hydroperoxides in cell membranes and is the major inhibitor of ferroptosis, which occurs in various neurological disorders with severe oxidative stress. The *GPX4* rs713041 C/T variant has been suggested to be most likely functional as having the ENCODE ranking score of 1b [56]. It has been proposed as an eQTL in blood and cerebellum and is not in linkage disequilibrium (LD) greater than 0.8 with any other variant [57]. This study found significantly lower frequency of the *GPX4* rs713041 T allele in PMS male patients compared to RRMS, suggesting it might have protective effects against disease progression. In a recent meta-analysis, the T allele was associated with a lower risk pre-eclampsia development and a higher risk of developing colorectal cancer (reviewed in [12]). In addition, the TT genotype was observed more frequently in patients with Alzheimer’s disease (AD) who exhibited normal long-term visual memory scores [30]. A different report from the Polish population found that the TT genotype and T allele were more frequent in MS patients compared to healthy controls, and when divided by sex the association remained significant only in males [47]. It is worth mentioning that the male study group in that study was much smaller than in current study, which was analyzing *GPX4* rs713041 C/T variant with regard to MS disease severity, not with occurrence of the disease. A possible explanation for this opposite result could lie in the C allele frequency of the Polish control group, which was much higher than that reported for the European population by GnomAD [31]. Regarding the possible effect of the rs713041 C/T variant on GPX4 activity, it was shown in Caco-2 cells (human colorectal adenocarcinoma) that in the selenium (Se)-deficient conditions cells overexpressing the C allele had higher ROS and lower GPX4 activity compared to those expressing the T allele [31]. Previous findings demonstrated that levels of Se in PMS patients were significantly lower compared to RRMS patients [58]. These observations may suggest that T allele confers a protective effect on disease severity, whereas the C allele may be associated with an increased risk of MS progression in patients with lower Se levels. In addition, no association of *GPX4* rs713041 C/T with *GPX4* mRNA level in PBMCs was observed, which is in concordance with previous studies that were analyzing effect of this variant on *GPX4* mRNA levels in Caco-2 cells and B lymphocytes [31,32]. Nevertheless, these findings require further validation and replication in larger cohorts of MS patients.

GSH is an essential substrate for GPX4 to suppress lipid peroxidation [59], and depletion of GSH has been implicated in different neurological diseases [60]. High GPX4 activity leads to a higher consumption of GSH, while GPX4 knockdown or inhibition can increase intracellular GSH levels [61]. Previously, we reported lower levels of total GSH and GSSG in the plasma of PMS patients compared to RRMS patients [27]. In the current study, we have found that plasma GPX4 was moderately positively correlated with GSH and the GSH/GSSG ratio in a subset of 100 MS patients. Recently, a very similar result was found in patients with community-acquired pneumonia, where serum GPX4 and the GSH/GSSG ratio were also moderately correlated (r  =  0.30) and their lower levels were independent risk factors for 30-day mortality [62]. The GSH/GSSG ratio reflects the intracellular redox balance and has been shown to have a close association with sensitivity to ferroptosis in cancer cells, but the molecular mechanism by which GSH could regulate ferroptosis are still not well studied [63]. The GSH/GSSG ratio in the MS study group was rather low, whereas the 75th percentile had a value of 1.55. In the EAE-induced rats, the reduction in the GSH/GSSG ratio was associated with a higher clinical score, oxidative damage, an increase in lipid peroxidation products and carbonylated proteins in the brain, spinal cord, and blood [64]. The lipid peroxidation marker 4-hydroxynonenal (4-HNE) was increased in the CNS of EAE mice [65] and in a model of progressive MS in mice [66]. In this study, the highest quartile of GSH/GSSG ratio in MS patients was significantly associated with lower plasma 4-HNE levels in the same group, confirming the presence of ferroptosis and that the GSH/GSSG ratio is a valid marker of redox state.

### 4.3. NQO1

NQO1 is involved in the cell protection against oxidative damage by detoxifying quinones, preventing the formation of harmful semiquinone radicals, and so limiting ROS production [47]. NQO1 is expressed in tissues that require robust antioxidant protection, including the CNS [67], where it is predominantly found in astrocytes and a subset of oligodendrocytes [68]. The *NQO1* rs1800566 variant contributes to the occurrence of neurodegenerative and neuroinflammatory disorders, like Alzheimer’s disease and MS [33]. The altered NQO1 protein has a distinctly reduced half-life, decreased stability and an ability to bind flavin adenine dinucleotide, which affect stability and activity of the enzyme [69]. Moreover, the lack of NQO1 protein expression was detected by Western blot on the post mortem brain sections of the AD patients and was correlated with the presence of *NQO1* rs1800566 rare allele homozygotes [34]. Authors suggested that NQO1 inactivation might be in correlation with AD occurrence [34]. This study found significantly lower *NQO1* mRNA level in genotypes containing the rare A allele compared to wild-type homozygotes in PBMC of MS patients. Nevertheless, the genotype frequency distribution of *NQO1* rs1800566 did not differ between RRMS and PMS patients. Studies up to now have investigated this variant only with occurrence of the MS, but not with its severity. MS patients from Greece had a higher frequency of AA genotype compared to controls [33]. The AA genotype frequency in Greek MS patients and in the patient group of the current study was similar, but no significant difference was observed between RRMS and PMS patients [33]. This could suggest that this variant may be a potential risk factor for occurrence of the disease but not to its severity. Though, *NQO1* rs1800566 has been investigated as a drug metabolizing marker [70,71], the data regarding association studies of this variant with the MS or other neurodegenerative diseases is scarce, so the discussion on this issue is limited.

### 4.4. CAT

Catalase is commonly expressed in the CNS, with predominant location in peroxisomes of astrocytes and oligodendrocytes [72], which are engaged in complex, dynamic interactions that dictate the progression of demyelination and the success of remyelination [73]. Gene variant *CAT* rs2420388 has been associated with the decreased catalase levels in the GWAS of the plasma proteome [49], while lower levels of catalase were associated with neurodegenerative diseases such as AD and Parikinson’s disease (reviewed in [35]). This variant has been proposed to be eQTL in blood, B and T cells [74], and is likely to be functional in brain, PBMCs, and CD4+ cells, as suggested by ENCODE database [56]. Nevertheless, the present study did not identify an association of this variant neither with PMS, nor with *CAT* mRNA levels in the PBMCs of MS patients. The variant also was not associated with diabetic nephropathy in the French population [75]. However, in this study, the rare allele containing genotypes were associated with lower plasma levels of GPX4 in MS patients overall. Glutathione peroxidases and catalase work together toward defense against ROS, with both of them decomposing hydrogen peroxide to water and oxygen but through different mechanisms [35]. Considering the role that catalase has in oxidative stress defense, it is not surprising that single nucleotide variant does not have a robust effect on its expression level, and that multiple mechanisms on different levels are engaged in its regulation. The proposed functionality of this variant needs to be analyzed in experimental mechanistic studies, and investigation of its possible regulatory effect needs to be conducted in a larger sample size than it was in this study.

### 4.5. Study Limitations

When analyzing MS disease severity rather than MS occurrence, it is always a challenge to achieve an equal number of patients in groups with mild (RRMS) and severe (PMS) form of the disease, which would be of benefit for the genetic association analysis. Even though MS primarily influences brain structures, the mRNA expression analysis was performed on PBMCs because of generally limited access to the human brain tissue and cerebrospinal fluid, and because it is minimally invasive, readily available sample type for research and diagnostics. Nevertheless, studies in post mortem brain tissue and in cerebrospinal fluid would give more specific insight into the direct link with the central nervous system-based pathophysiology of multiple sclerosis. In addition, the results regarding the effect of variants on mRNA and circulatory biomarkers levels need to be validated in larger sample groups. The analysis of mRNA expression with regard to genotypes within different patient groups, RRMS and PMS, was not performed due to the limited sample size used for RNAseq and consequently lower study power. Although the variants were meticulously selected to have functional effects in tissues susceptible to MS according to available databases, that effect could not be verified for every variant. Moreover, the potential effects of other variants in LD with the selected ones cannot be ruled out, nor can higher study power achieved through haplotype analysis. Aside from the rather limited sample size, another possible explanation is that their effect differs substantially between cell types, considering the impact of oxidative stress and ferroptosis on cells. In addition, validation on protein level, considering the effect of post-transcriptional and post-translational regulation on mRNA, would have strengthened the functional interpretation of the findings. Since analysis at the protein level was not performed, it should be investigated in the future studies.

## 5. Conclusions

These findings depict the effect of selected variants in genes crucial for antioxidant defense and ferroptosis on MS disease severity. The results indicate that, among the five investigated variants, *GCLC* rs572496 has the most potential for further research, as it was significantly associated with MS disease severity and had a significant and independent effect on *GCLC* mRNA levels in PBMCs of MS patients. The *NQO1* rs1800566 had significant and independent effect only on *NQO1* mRNA level in PBMCs. In addition, *GPX4* rs713041 showed a sex-specific association with MS severity in male patients. Further comprehensive studies are required to elucidate the proposed functionality of these variants and to validate these results in larger, independent cohorts focusing on MS disease severity.

## Figures and Tables

**Table 1:** The main information on selected gene variants according to the inclusion criteria.

Gene	Variant (rs)	Allelic Change	Chromosome	Variant Type	MAF	eQTL (Tissues)	RegulomeDB Score	GWAS Evidence	Variants in LD (n)
*GCLC*	rs572496	C/T	chr6	intronic	0.46	brain, heart, artery	1f	No	14
*GCLM*	rs2273406	G/A	chr1	intronic	0.17	brain, immune cells, artery, heart	1f	No	32
*GPX4*	rs713041	C/T	chr19	synonymous	0.45	blood, cerebellum	1b	No	none
*NQO1*	rs1800566	A/G	chr16	missense	0.19	blood	1b	Yes (NADPH level)	>100
*CAT*	rs2420388	A/G	chr11	intronic	0.23	blood, T cells, B cells	1f	Yes (catalase level)	21

MAF—minor allele frequency; eQTL—expression quantitative trait loci; LD—linkage disequilibrium, GWAS—genome wide association study.

**Table 2:** Basic characteristics of MS patients divided with regard to the MS severity in RRMS and PMS groups, involved in the analysis of selected gene variants.

Characteristics	RRMS n = 604	PMS (SPMS + PPMS) n = 241	*p*
Age, years	38.86 ± 10.41	47.27 ± 9.93	<0.01 ^a^
Sex, f/m, %	0.61/0.39	0.64/0.36	0.53 ^b^
Smoking, %	0.33	0.36	0.60 ^b^
Disease duration, years	7.73 ± 5.83	14.07 ± 9.44	<0.01 ^c^
EDSS	2.47 ± 1.32	5.59 ± 1.56	<0.01 ^c^
MSSS	3.91 ± 2.35	6.52 ± 2.03	<0.01 ^c^
gARMSS	4.58 ± 2.23	7.17 ± 2.01	<0.01 ^c^

Continual parameters are presented as mean ± standard deviation (SD); RRMS—relapsing–remitting multiple sclerosis; PMS—progressive multiple sclerosis (SPMS + PPMS); SPMS—secondary progressive multiple sclerosis; PPMS—primary progressive multiple sclerosis; Age—age at blood sampling; EDSS—Expanded Disability Status Scale; MSSS—Multiple Sclerosis Severity Score; gARMSS—Age-Related Global MS Severity Score; ^a^—Student’s *t* test; ^b^—chi-square test; ^c^—Mann–Whitney U test. *p* values < 0.05 are considered statistically significant.

**Table 3:** Genotype and allele frequency distribution of investigated gene variants in RRMS and PMS patients.

Gene/Locus	Gene Variant	RRMS, n (%)	PMS, n (%)	*p*
		n = 604	n = 241	
*GCLC*	rs572496			
	CC	217 (0.36)	89 (0.37)	
	CT	278 (0.46)	128 (0.53)	0.02
	TT	109 (0.18)	24 (0.10)	
	CC + CT	495 (0.82)	217 (0.90)	0.007
	TT	109 (0.18)	24 (0.10)	
	allele T	0.41	0.37	0.08
*GCLM*	rs2273406			
	GG	321 (0.66)	132 (0.72)	0.11
	GA + AA	225 (0.34)	91 (0.28)	
	allele A	0.19	0.15	0.06
*GPX4*	rs713041			
	TT	114 (0.19)	40 (0.16)	
	TC	338 (0.56)	127 (0.53)	0.25
	CC	152 (0.25)	74 (0.31)	
	allele T	0.47	0.43	0.14
	Males	n = 236	n = 86	
	TT + TC	184 (0.78)	58 (0.67)	0.05
	CC	52 (0.22)	28 (0.33)	
	allele T	0.49	0.38	0.02
*NQO1*	rs1800566			
	GG	399 (0.66)	161 (0.67)	
	GA	175 (0.29)	70 (0.29)	0.89
	AA	30 (0.05)	10 (0.04)	
	allele A	0.19	0.19	0.71
*CAT*	rs2420388			
	GG	320 (0.53)	133 (0.55)	
	GA	242 (0.40)	96 (0.40)	0.55
	AA	42 (0.07)	12 (0.05)	
	allele A	0.27	0.25	0.38

*p*—chi-square test; *p* values < 0.05 are considered significant. *GCLM* rs2273406 genotypes are shown according to the GG vs. GA + AA model, since the AA genotype was present in only 4 PMS patients, and the chi-square test is inaccurate when expected numbers are less than 5.

**Table 4:** Correlations of GPX4 with circulatory molecular indicators of glutathione-related antioxidant defense.

	R	*p*	Adjusted R *	Adjusted *p* *
GPX4	1			
GSH	0.30	0.003	0.29	0.005
GSSG	−0.26	0.009	−0.18	0.89
GSH/GSSG	0.34	0.001	0.31	0.003

* adjusted for age, sex, disease course and disease duration. R—Spearman’s rank correlation coefficient. *p* values < 0.05 were considered statistically significant.

## Data Availability

The original contributions presented in the study are included in the article; further inquiries can be directed to the corresponding author.

## Supplementary Materials

The following supporting information can be downloaded at: https://www.mdpi.com/article/10.3390/biology15100773/s1, Figure S1: Scatter plot with regression line showing Spearman’s correlation between GPX4 and (A) GSH, (B) GSSG and (C) GSH/GSSG; Table S1: *GCLC* rs572496 effect on *GCLC* mRNA expression adjusted for clinical and demographic variables; Table S2: *NQO1* rs1800566 effect on *NQO1* mRNA adjusted for clinical and demographic variables; Table S3: Association of investigated gene variants with circulatory molecular indicators of glutathione-related antioxidant defense in MS patients.

## Abbreviations

The following abbreviations are used in this manuscript:

MS	multiple sclerosis
ROS	reactive oxygen species
GSH	glutathione
CNS	central nervous system
GCL	glutamate cysteine ligase
GPX4	glutathione peroxidase 4
GSSG	oxidized glutathione
GR	glutathione reductase
NQO1	NAD(P)H quinone dehydrogenase 1
CAT	catalase
PPI	protein–protein interactions
SeCys	selenocysteine
RRMS	relapsing–remitting multiple sclerosis
PMS	progressive multiple sclerosis
SPMS	secondary progressive multiple sclerosis
PPMS	primary progressive multiple sclerosis
EDSS	expanded disability status scale
MSSS	multiple sclerosis severity score
gARMSS	age-related global multiple sclerosis severity score
DEGs	differentially expressed genes
MAF	minor allele frequency
4PL	four-parameter logistic standard curve
OD	optical density
OR	odds ratio
CI	confidence interval
SD	standard deviation
AD	Alzheimer’s disease
AOC	antioxidative capacity levels
