# Supplementary material for: Association Between Multiple Sclerosis Severity and Functional Variants in Key Antioxidant Defense and Ferroptosis-Related Genes
Source: Biology (Basel). 2026 May 12;15(10):773. doi: 10.3390/biology15100773 (PMC13203362; doi:10.3390/biology15100773)
Supplement: Supplementary file 1 [file biology-15-00773-s001.zip › biology-4236933-supplementary.pdf]

Association Between Multiple Sclerosis Severity and Functional Variants in Key Antioxidant Defense and Ferroptosis-Related Genes

Tamara Djuric [1,*], Jovana Kuveljic [1], Ana Djordjevic [1], Milan Stefanovic [1], Evica Dincic [2,3], Mariana Seke [1], Aleksandra Stankovic [1] and Maja Zivkovic [1]

Table S1. *GCLC* rs572496 effect on *GCLC* mRNA expression adjusted for clinical and demographic variables

Predictors	Beta	SE	p
Age	-0.06	0.35	0.86
Sex	0.24	0.15	0.11
Disease course	1.01	0.72	0.19
Disease duration	-0.53	0.26	0.05
EDSS	2.29	1.81	0.21
MSSS	-1.88	0.78	0.02
gARMSS	-1.02	1.53	0.51
GCLC rs572496 CC vs. CT+TT	0.31	0.14	0.03

Multiple linear regression analysis. EDSS -Expanded Disability Status Scale; MSSS - Multiple Sclerosis Severity Score; gARMSS - Age-Related global Multiple Sclerosis Severity; Beta – standardized beta coefficient; SE – standard error.

Table S2. *NQO1* rs1800566 effect on *NQO1* mRNA adjusted for clinical and demographic variables

Predictors	Beta	SE	p
Age	-0.84	0.35	0.02
Sex	0.08	0.15	0.56
Disease course	-0.40	0.70	0.57
Disease duration	-0.15	0.26	0.57
EDSS	5.80	1.77	0.00
MSSS	-1.18	0.77	0.14
gARMSS	-4.42	1.50	0.01
NQO1 rs1800566 GG vs. GA+AA	-0.38	0.13	0.006

Multiple linear regression analysis EDSS -Expanded Disability Status Scale; MSSS - Multiple Sclerosis Severity Score; gARMSS - Age-Related global Multiple Sclerosis Severity; Beta – standardized beta coefficient; SE – standard error.

Table S3. Association of investigated gene variants with circulatory molecular indicators of glutathione-related antioxidant defense in MS patients

Parameter	*GCLC* rs572496		
	CC	CT+TT	p
GSH (μmol/l)	17.23±7.47	15.34±7.67	0.09
GSSG (μmol/l)	13.94±2.59	13.71±3.14	0.81
GSH/GSSG	1.32±0.73	1.19±0.68	0.27
GPX4 (pg/ml)	2565.97±1523.29	2465.66±1512.15	0.34
	GCLM rs2273406		
	GG	GA+AA	p
GSH (μmol/l)	16.30±8.45	15.85±7.22	0.74
GSSG (μmol/l)	13.40±2.04	13.97±3.30	0.45
GSH/GSSG	1.26±0.71	1.23±0.69	0.92
GPX4 (pg/ml)	2367.29±1307.21	2534.02±1601.82	0.90
	GPX4 rs713041		
	TT+TC	CC	p
GSH (μmol/l)	15.53±7.13	17.33±8.82	0.49
GSSG (μmol/l)	14.01±3.10	13.11±2.29	0.20
GSH/GSSG	1.19±0.67	1.38±0.77	0.22
GPX4 (pg/ml)	2414.89±1385.70	2725.98±1808.88	0.24
	NQO1 rs1800566		
	GG	GA+AA	p
GSH (μmol/l)	15.75±7.22	16.52±8.47	0.98
GSSG (μmol/l)	14.13±2.88	12.99±2.95	0.15
GSH/GSSG	1.17±0.56	1.41±0.92	0.71
GPX4 (pg/ml)	2517.16±1584.05	2457.21±1354.97	0.47
	CAT rs2420388		
	GG	GA+AA	p
GSH (μmol/l)	16.50±7.92	15.42±7.31	0.64
GSSG (μmol/l)	13.40±2.68	14.29±3.14	0.19
GSH/GSSG	1.31±0.73	1.16±0.66	0.37
GPX4 (pg/ml)	2736.80±1680.85	2180.05±1218.09	0.03

Values of continuous parameters are presented as mean ± SD; GSH – reduced glutathione; GSSG – oxidized glutathione; p —Student's t test; p - values < 0.05 were considered statistically significant.

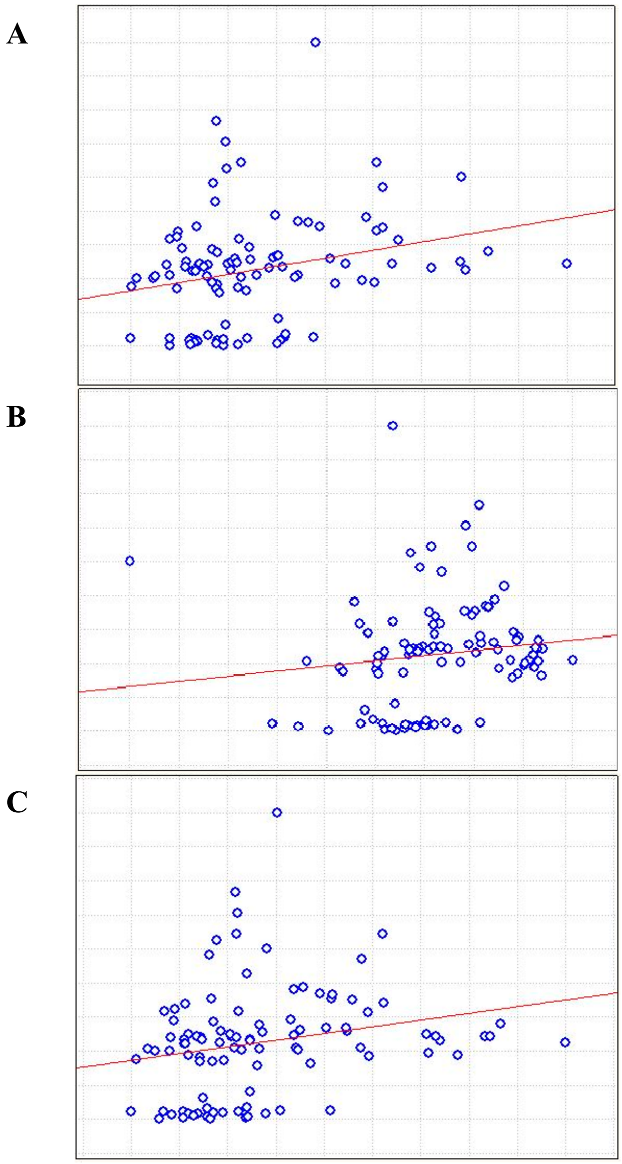

Figure S1. Scatter plot with regression line showing Spearman`s correlation between GPX4 and **A)** GSH, **B)** GSSG and **C)** GSH/GSSG.
